# Effects of silver nanoparticles on the physiology, stress, and mineral uptake of banana cultivars *in vitro* and greenhouse

**DOI:** 10.3389/fpls.2025.1527137

**Published:** 2025-08-12

**Authors:** Natalia Mendoza, Karen Hidalgo, Lorena Troya, Eduardo Sánchez-Timm, Joel Vielma, María Eulalia Vanegas, Nina Bogdanchikova, Alexey Pestryakov, Pablo Chong

**Affiliations:** ^1^ ESPOL Polytechnic University, ESPOL, Facultad de Ciencias Naturales y Matemáticas, Departamento de Ciencias Químicas y Ambientales, Campus Gustavo Galindo, Guayaquil, Ecuador; ^2^ ESPOL Polytechnic University, Escuela Superior Politécnica del Litoral, Centro de Investigaciones Biotecnológicas del Ecuador, Guayaquil, Ecuador; ^3^ ESPOL Polytechnic University, Escuela Superior Politécnica del Litoral, Facultad de Ciencias de la Vida, Guayaquil, Ecuador; ^4^ N@NO-CEA GROUP (Center for Environmental Studies), Department of Applied Chemistry and Production Systems, Faculty of Chemical Sciences, University of Cuenca, Cuenca, Ecuador; ^5^ Universidad Nacional Autónoma de México, Centro de Nanociencias y Nanotecnología, Ensenada, BC, Mexico; ^6^ Research School of Chemistry and Applied Biomedical Sciences, Tomsk Polytechnic University, Tomsk, Russia

**Keywords:** silver nanoparticles, banana, plant minerals uptake, gene expression, greenhouse conditions

## Abstract

**Introduction:**

This study explores the effects of silver nanoparticles (AgNPs) from the formulation Argovit™ on physiological stress responses and mineral uptake in banana cultivars, both *in vitro* and under greenhouse conditions. These specific AgNPs have been previously studied for their antifungal activity against *Fusarium oxysporum*, highlighting their potential as a disease control agent in banana cultivation. Evaluating their phytotoxicity is crucial to determine safe application levels, particularly at the concentrations previously shown to be effective.

**Methods:**

The primary objective is to expose the phytotoxic effects, nutrient uptake, and translocation mechanisms of AgNPs based on their application method, either foliar or drench.

**Results and discussion:**

*In vitro* experiments on the Cavendish banana var. Williams, with shoots cultured in media supplemented with AgNPs at concentrations of 0, 25, 50, 100, and 1000 mg L^-1^, showed significant reductions in shoot formation, length, chlorophyll content, and leaf number as AgNP concentrations increased. Rooting experiments revealed similar trends with high AgNP concentrations resulting in a decreasing root number and size. Greenhouse experiments on Gros Michel bananas, evaluating AgNP uptake through foliar and drench applications at 0, 25, 50, and 100 mg L^-1^, monitored over a month, showed no statistically significant differences in growth parameters between treated plants and controls. However, tissue analysis revealed higher leaf Ag concentrations than roots and stems. The study also analyzed antioxidant gene expression via qPCR, targeting genes such as *MaSOD* (superoxide dismutase), *MaCAT* (catalase)*, MaAPX* (ascorbate peroxidase), and *MaGPX* (glutathione peroxidase), showing altered profiles in response to AgNP exposure and indicating induced oxidative stress. This research underscores the complex interactions between AgNPs and banana plants, emphasizing the need for further study to optimize safe and effective AgNP application in agriculture, balancing crop protection and environmental safety.

## 1 Introduction

Bananas (*Musa* species) are among the most important and nutrient-rich crops worldwide, valued for their economic significance and global consumption, particularly in Ecuador and other major production regions (FAO, 2023). However, the banana industry faces significant challenges due to established and emerging pathogens, which threaten its sustainability and economic viability. *Fusarium oxysporum* tropical race 4 (TR4) is a major pathogen that poses a direct threat to banana production worldwide (Ordonez et al., 2015; Ploetz et al., 2015; Kema et al., 2021; Risède et al., 2010). Similarly, black Sigatoka, caused by *Pseudocercospora fijiensis*, further endangers the industry’s future (Chong et al., 2021; Voora et al., 2023).

The European Union (EU) has raised concerns over the intensive use of agrochemicals in banana cultivation, citing environmental, health, and regulatory factors as critical issues. Banana production often relies heavily on pesticides, with application rates surpassing many other crops, creating risks for farmworkers and surrounding ecosystems through pesticide runoff and contamination. The EU’s Green Deal objectives emphasize the need for sustainable agriculture to reduce chemical inputs and mitigate agriculture’s environmental impact (Elbehri et al., 2016; From Science to Field Banana Case Study-Guide Number 1, n.d.; Laprade, 2023).

Given these challenges, there is an urgent need to explore sustainable alternatives for managing banana pathogens. Nanotechnology has revolutionized several scientific fields, and silver nanoparticles (AgNPs) are gaining research attention for their potential to enhance crop protection, their unique physicochemical properties (Joudeh and Linke, 2022), and antimicrobial activity (More et al., 2023). Operating at the nanoscale, this technology demonstrates its ability to address the challenges prevalent in conventional agriculture through meticulous design, engineering interventions, and innovative approaches. Its adaptability to desired dimensions further underscores its potential to revolutionize agricultural practices (Santás-Miguel et al., 2023; Kamal et al., 2023). The small size gives the AgNPs more catalytic and biochemical activity, allowing them to be easily transported and introduced into biological systems through cell walls and membranes (Li et al., 2022).

Several studies have demonstrated the broad-spectrum antifungal activity of AgNPs against numerous phytopathogens. Studies have shown that AgNPs inhibit the growth of fungi such as *Botrytis cinerea*, *Alternaria alternata*, *Colletotrichum gloeosporioides*, *Phomopsis* spp., and *Fusarium* spp. through multiple mechanisms, including disruption of cell membrane integrity, generation of reactive oxygen species (ROS), inhibition of hyphal growth, and interference with spore germination and energy metabolism (Shi et al., 2023; Fallah et al., 2024; Rami et al., 2024; Tabassum et al., 2024; Mendoza et al., 2025).

Moreover, synergistic effects have been observed when AgNPs are combined with conventional fungicides like azoxystrobin, enhancing their antifungal efficacy while potentially reducing the required chemical dosage (Osman et al., 2022). These findings underline the potential of AgNPs as promising alternatives or complements to traditional fungicides in sustainable agriculture.

For this reason, AgNPs represent a promising and innovative alternative to traditional agrochemicals, with potential benefits for crop protection (Matras et al., 2022; Osman et al., 2022), food safety, and productivity (Mansoor et al., 2021; Nandini et al., 2023). Despite this, the impact of AgNPs on plant health and development is still poorly understood. This has caused concern in the scientific society, given that the unknown long-term effects and potential toxicity of AgNPs could pose unforeseen risks to plants (Vishwakarma et al., 2017), ecosystems (Tortella et al., 2020), and human health (Noga et al., 2023).

Scientific advances focus on developing more stable, eco-friendly nanoparticles for agriculture. Recent studies on biosynthesized AgNPs from plant extracts aim to enhance their stability and properties for field applications. These nanoparticles exhibit superior bioactivity, influencing plant stress responses, nutrient uptake, and pollutant degradation. Their effectiveness depends on the dispersion medium, making stability a key factor in agricultural use (Asif et al., 200AD; Ihsan et al., 2021; Habib et al., 2024).

As a result, further comprehensive studies are necessary to fully explain the benefits and potential drawbacks of using AgNPs in agriculture. Besides, it is unclear whether the AgNPs can be distributed in the plant when applied by spraying or irrigation. Previous reports have shown that AgNPs improve seed germination in watermelon crops (*Citrullus lanatus*) (Acharya et al., 2020), plant growth of *Oryza sativa* at 30 μg mL^-1^ (Sondi and Salopek-Sondi, 2004; Khan et al., 2023).

Our previous research demonstrated that these formulations of AgNPs were effective in controlling *Fusarium oxysporum* f.sp. *cubense* (Foc) under both *in vitro* and greenhouse conditions (Mendoza et al., 2025). However, for their practical application in agricultural settings, it is essential to determine whether AgNPs are safe for plants and do not cause unintended physiological effects, particularly at concentrations of 25–100 mg L^-1^, where they work as an antifungal. Understanding their impact on plant health, nutrient uptake, and translocation will help evaluate their feasibility as a sustainable crop protection strategy.

This study aims to assess the potential toxicity of AgNPs in the selected doses on banana plants by analyzing their physiological effects, nutrient dynamics, and distribution patterns within plant tissues. This research contributes to a more comprehensive understanding of AgNPs’ safety and applicability in sustainable agriculture by addressing these critical aspects.

## 2 Materials and methods

### 2.1 AgNP synthesis and formulations

Argovit-1220 AgNP was formulated based on Argovit commercial formulation with different stabilizers (**Supplementary Table S1**). Argovit formulation was obtained from the Scientific-Production Centre Vector-Vita Ltd (Novosibirsk, Russia). Argovit is an aqueous suspension of AgNPs with an average size of 38 ± 15 nm stabilized by a polyvinylpyrrolidone (PVP) coating. The metallic content of Ag is 12 mg mL^-1^ with 188 mg mL^-1^ of PVP to a final concentration of AgNPs 200 mg mL^-1^ suspension. All stocks and aliquots were stored in the dark at 4°C.

### 2.2 *In vitro* Assays in Cavendish banana

#### 2.2.1 Effects of AgNPs on shoot multiplication

*In vitro* Cavendish banana (var. Williams) shoots, each measuring approximately 1 cm, were transferred to a multiplication medium containing: MS basal salts (from Sigma-Aldrich, Massachusetts, USA); 30 g L^-1^ sucrose, 50 mL L^-1^ coconut water, 0.52 mg L^-1^ Indole-3-Acetic Acid (IAA), and 1.75 mg L^-1^ kinetin, 4.0 mg L^-1^ Benzylaminopurine (BAP) and supplemented with 5 different AgNPs concentrations (0, 25, 50, 100, 1000 mg L^-1^). The pH of the medium was adjusted to 5.8 before dispensing it in culture flasks and autoclaving.

Cultures were organized in a randomized block design with five replicates per treatment, each containing ten explants per culture flask, which were incubated at 26°C for a 16-hour photoperiod for four weeks. Following the culture period, the shoot length (cm), chlorophyll (CCI), and the number of leaves developed were determined.

#### 2.2.2 Effects of AgNPs on rooting

*In vitro* banana plantlets obtained from the multiplication stage with the application of AgNPs were excised to a length of approximately 1 cm and subsequently transferred to the rooting medium. This medium comprised MS basal salts, 30 g L^-1^ sucrose, 50 mL L^-1^ coconut water, 0.26 mg L^-1^ IAA, and 0.86 mg L^-1^ kinetin, applying the same concentrations of AgNPs as previously mentioned. Cultures were arranged in five replicates per treatment, each flask contained ten explants, which were incubated under the same conditions as described above. The number of roots and the largest root length (cm) were recorded after four weeks.

### 2.3 Greenhouse assays in Gros Michel banana

A total of 80 Gros Michel plants, three months old and in the vegetative stage F2, were used in this experiment. Ten plants were assigned to each AgNP concentration and application method. The AgNP concentrations evaluated were 0, 25, 50, and 100 mg L^-1^, administrated by two application methods: foliar and drench. Of the ten plants assigned, five replicates were designated for the evaluation of uptake of AgNPs and physiological impact measurements, and the remaining five for gene expression evaluations, ensuring distinct sample groups. For the gene expression analysis, an incision was made on the leaf to induce stress. Separating these groups minimized any interference with physiological measurements, ensuring clarity in the data collected.

Stock solutions of AgNPs were prepared with AgNPs and 18.2% PVP at a concentration of 12,000 mg L^-1^. The stock solution was diluted with distilled water for both application methods to achieve the desired AgNP concentrations. The foliar application included 1% Triton in the solutions to enhance solution adherence to the leaves. AgNP solutions were applied weekly. The solutions were sprayed on both the adaxial and abaxial sides of the leaf for foliar applications, while for drench applications, 100 mL of each solution was poured near the pseudo-stem, approximately 5 centimeters from the substrate.

#### 2.3.1 Evaluation of AgNP physiological impact

The experiment spanned five weeks, during which various physiological parameters, including growth parameters and chlorophyll concentrations, were measured weekly. Growth parameters assessed were plant height, stem diameter, foliar emission, and leaf chlorophyll concentration. They were recorded at the start of the assay and after each weekly AgNP application. Leaf chlorophyll concentrations were measured with an Apogee chlorophyll concentration meter model MC-100 (Apogee Instruments, INC., Utah, USA) (Ling et al., 2011). At the end of the evaluation period, leaf tissue samples were collected to measure chlorophyll a, b, and total. In addition, each plant was weighed, and the length and number of roots were registered. Plants were dissected into three segments—leaves, roots, and stems— and dried thoroughly in an oven set at 65°C for three days. This drying process prepared the samples for subsequent analysis of AgNP absorption and elemental analysis in each tissue.

#### 2.3.2 Elemental analysis of plant tissues for Ag and mineral nutrients

Aerial parts (leaves and stems) were washed with deionized water (DW), whereas roots were washed with tap and DW, and subsequently immersed in a 0.01 M EDTA solution for 60 sec, followed by rinsing with DW. Roots, stems, and leaves were dried at 65°C for 48 h, and dry biomass was measured. Dried tissues were ground and passed to a 850 μm mesh to determine silver (Ag) and other elements in each plant part. The element analysis covered: zinc (Zn), copper (Cu), iron (Fe), magnesium (Mg), potassium (K), nitrogen (N), phosphorus (P), sulfur (S), calcium (Ca), boron (B), manganese (Mn), and molybdenum (Mo). In brief, 500 mg of pulverized dry biomass were mineralized in a microwave-assisted digestor (Ethos Up, Milestone, Italy) with 4 mL of HNO_3_ at two ramp temperatures, 120°C and 200°C for 20 and 10 min, respectively. After digestion, samples were diluted to 50 mL with DW and passed through a 0.45 μm filter paper. Mineral nutrients and Ag in the filtrates were determined by Inductively Coupled Plasma Optical Emission Spectrometer (ICP-OES, Avio 550, Perkin Elmer, Shelton, Connecticut, US).

### 2.4 Evaluation of gene expression exposed to AgNP concentrations

#### 2.4.1 RNA extraction protocol

For the RNA extraction, five plants per application group were selected at each AgNP concentration (0, 25, 50, and 100 mg L^-1^) and at four time points (0h, 6h, 12h, and 24h) for both application methods. A sample (approx. 100 mg) was taken from the third leaf of each plant, crushed with liquid nitrogen, and subjected to RNA extraction following the Ecuadorian Biotechnology Research Center-CIBE protocol established (CBE-PROT-BM-017).

Following trituration, 750 µL of extraction buffer (NaCl 400 mM, Tris-HCl 100 mM, EDTA 10 mM, PVP 40,000 at 2%, 50 µL of β-Mercaptoethanol, and water up to 5 mL) was added and mixed by the vortex. SDS (30%) was added to reach a final concentration of 2%, along with Proteinase K (final concentration of 350 µg). The mixture was incubated at 55°C for one hour, then centrifuged at 12,000 rpm at 4°C for 30 minutes. The supernatant (SN) was transferred to a new Eppendorf tube, mixed with an equal volume of Phenol: Chloroform: Isoamyl alcohol (25:24:1), and vortexed. Then, it was centrifuged at maximum speed at 4°C for 5 min and repeated the steps since the addition of Phenol: Chloroform: Isoamyl alcohol (25:24:1).

After the final centrifugation, the SN was collected, mixed with half its volume of 6M LiCl, and allowed to precipitate overnight at -20°C. The next day, samples were centrifuged at 12,000 rpm at 4°C for 30 min, the SN was discarded, and the RNA pellet was washed three times with 70% ice-cold ethanol. Finally, RNA was dissolved in 50 µL of RNase-free H_2_O and stored at -80°C.

Before future use, RNA quality was measured via Nanodrop (Thermo Scientific, Waltham, Massachusetts, USA), selecting three samples per group, based on 260/230 and 260/280 absorbance ratios (between 1.8 and 2.1). All RNA samples were adjusted to a concentration of 120 ng µL^-1^ for consistency in subsequent analyses.

#### 2.4.2 cDNA synthesis

The retrotranscription process was performed using ABScriptII One Step RT-qPCR kit (ABclonal, Woburn, Massachusetts, USA) enzyme in combination with the reaction buffer of the Maxima first strand cDNA kit (Thermo Scientific, Waltham, Massachusetts, USA). Before retrotranscription, DNase treatment was applied using the RQ1 RNase-free DNase kit (Promega, Madison, Wisconsin, USA) to eliminate any residual DNA contamination that could interfere with future analyses.

Calculations were performed to ensure that final cDNA concentrations were as consistent as possible to achieve uniformity across samples. To verify the absence of DNA contamination, endpoint PCR was conducted post-transcription using Actin primers (FWD ACAGTGTCTGGATTGGAGGC and REV GCACTTCATGTGGTGGACAATGG), confirming the presence of cDNA only and ensuring sample purity for downstream applications.

#### 2.4.3 Gene expression analysis of antioxidant-related genes using quantitative real-time PCR

qPCR was conducted on the cDNA synthesized in previous steps to analyze expression variations in the antioxidant-related genes, including *MaSOD*, which encodes the enzyme superoxide dismutase*; MaCAT*, which encodes the enzyme catalase*; MaAPX*, which encodes the enzyme ascorbate peroxidase*; and MaGPX*, which encodes the enzyme glutathione peroxidase. These genes, as previously reported (Qian et al., 2013) exhibit altered expression in the presence of silver nanoparticles. Gene expression levels were compared against the housekeeping gene *Actin*. Primers used for each gene are listed in **Table 1**.

**Table 1:** Primers used in this study for each one of the analyzed genes.

Gene	FWD	REV
*MaSOD*	GCACTTCATGTGGACAATGG	CAGAGCTAGCCACTGACATAAG
*MaCAT*	ACGACTTCGACCCGACCCGCTCGATGA	ACGCTAGCTGCTCGCTCGTTCTCCGA
*MaGPX*	TCGTCCCCTGCTCCTCCTTCTTGCT	CCCATGGCATCATCCTTGACGGTGA
*MaAPX*	ACTTCTGTTGTGCCGATCTC	CTGCTCTCTCGGGAAGCTTTATC
*Actin*	ACAGTGTCTGGATTGGAGGC	GCACTTCATGTGGTGGACAATGG

The qPCR reactions were prepared with the GoTaq qPCR Master Mix (Promega, Madison, Wisconsin, USA), following the manufacturer’s instructions, and carried out in the thermal cycler for real-time PCR, Quant Studio Real-Time PCR Instrument (Thermo Scientific, Waltham, Massachusetts, USA). The cycle threshold (Ct) values obtained during qPCR served as the primary metric for quantifying gene expression. These Ct values were subsequently used to calculate relative quantification and fold change, based on the following process:


$$\Delta C_{t}\;sample_{1}\;={\text{ C}}_{t}\text{gene test}-{\text{C}}_{t}\text{endogenous control }$$

$$\;\Delta \Delta C_{t}=\;\Delta {\text{C}}_{t}{\text{ sample}}_{1}-\;\Delta C_{t}calibrator$$

$$\text{Relative Quantification }=\;2^{-\Delta \Delta {\text{C}}_{t}}$$

$$\text{Fold change }=\log_{2}(\text{RQ})$$

### 2.5 Statistical analysis

Data were analyzed by an analysis of variance (ANOVA) carried out to test the differences in the plants’ effects. The statistical significance of differences between mean values was determined using different *post hoc* tests with p < 0. 05. Statistical analysis was performed using the R software (ver. 4.4.1, Posit Software, PBC, Boston, Massachusetts, USA) (Posit Team, 2024).

## 3 Results

### 3.1 *In vitro* assays in Cavendish banana

**Figure 1:**
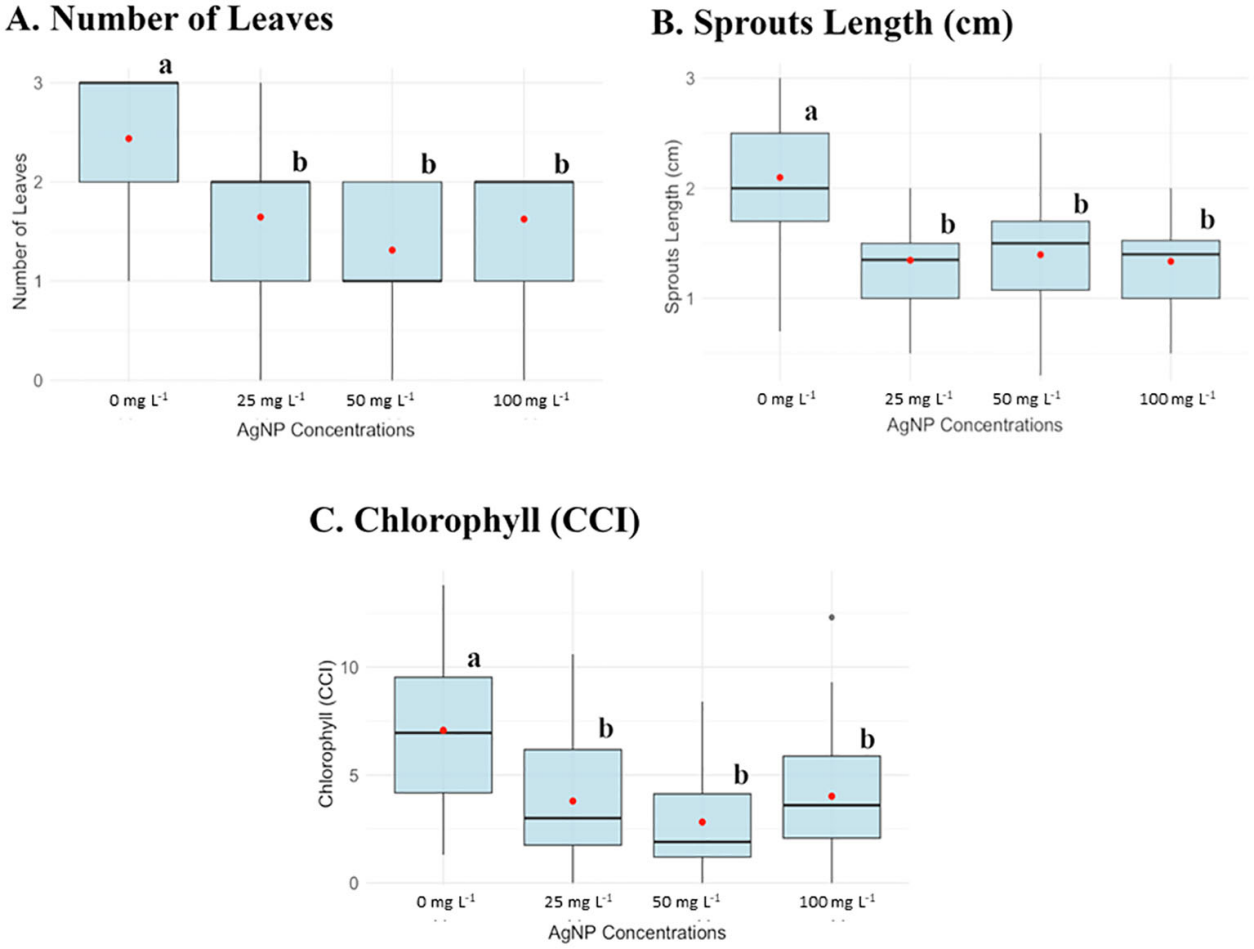
Box plots of parameters evaluated in the *in vitro* experiment of AgNPs on shoot multiplication at different AgNP concentrations: 0, 25, 50, and 100 mg L^-1^. **(A)** Number of leaves, **(B)** Sprouts length (cm), and **(C)** Chlorophyll content (CCI). Different letters (a, b) indicate statistically significant differences between groups of concentrations according to the Kruskal-Wallis test (p < 0.05).

**Figure 2:**
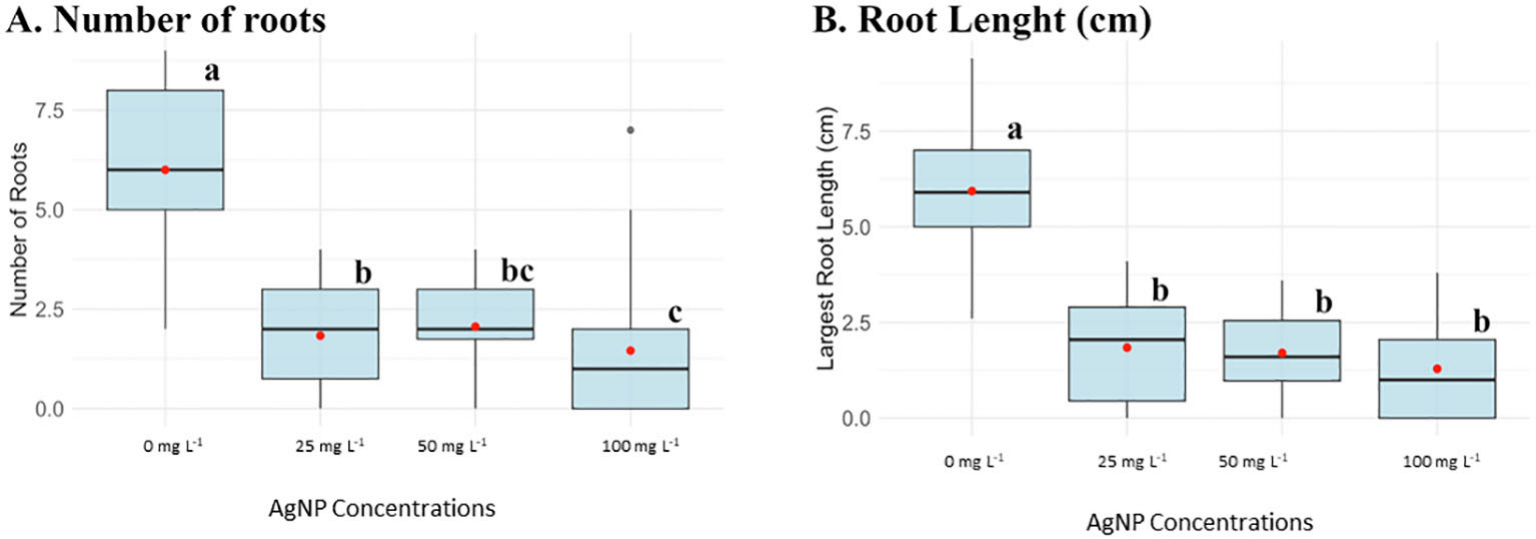
Box plots of parameters evaluated in the *in vitro* experiment of AgNPs on shoot multiplication at different AgNP concentrations: 0, 25, 50, and 100 mg L^-1^. **(A)** Number of roots and **(B)** Root length (cm). Different letters (a, b, c, bc) indicate statistically significant differences between groups of concentrations according to the Kruskal-Wallis test (p < 0.05). Groups sharing at least one letter are not significantly different from each other.

Data analysis shown in **Figures 1**, **2** indicated a significant reduction in all measured parameters in the presence of the AgNPs compared to the control group (0 mg L^-1^ AgNPs), as confirmed by post-Anova tests. However, no statistically significant differences were observed between AgNP-supplemented plants at concentrations of 25, 50, and 100 mg L^−1^, except for root number, where each concentration showed significant differences.

Plants without nanoparticles had an average leave number of 2.5, while plants supplemented with AgNPs exhibited a reduced leave number ranging from 1 to 1.5. Similarly, the average shoot length in the control group was 2 cm but decreased to 1 cm with AgNP, indicating a 50% reduction in size. Chlorophyll content (CCI) also declined with AgNP, the control plants averaged a CCI of 6, whereas plants supplemented with AgNP had a CCI range of 1–2 (**Figure 1**). Besides, at the concentration of 1000 mg L^-1^, none of the parameters were measured because this concentration was lethal for the banana plant to develop.

AgNPs were added to the rooting culture media at concentrations of 25, 50, and 100 mg L^−1^, and root development was also substantially affected by AgNPs. **Figure 2** showed that control plants had an average of six roots, which decreased to a range of 1.5–2 roots in AgNP-treated plants. Additionally, the largest root length normally averaged 6 cm in the absence of nanoparticles and, across all AgNP concentrations, was reduced to 1 cm.

### 3.2 Greenhouse assays in Gros Michel banana

#### 3.2.1 Physiological analysis

##### 3.2.1.1 Foliar application

**Figure 3:**
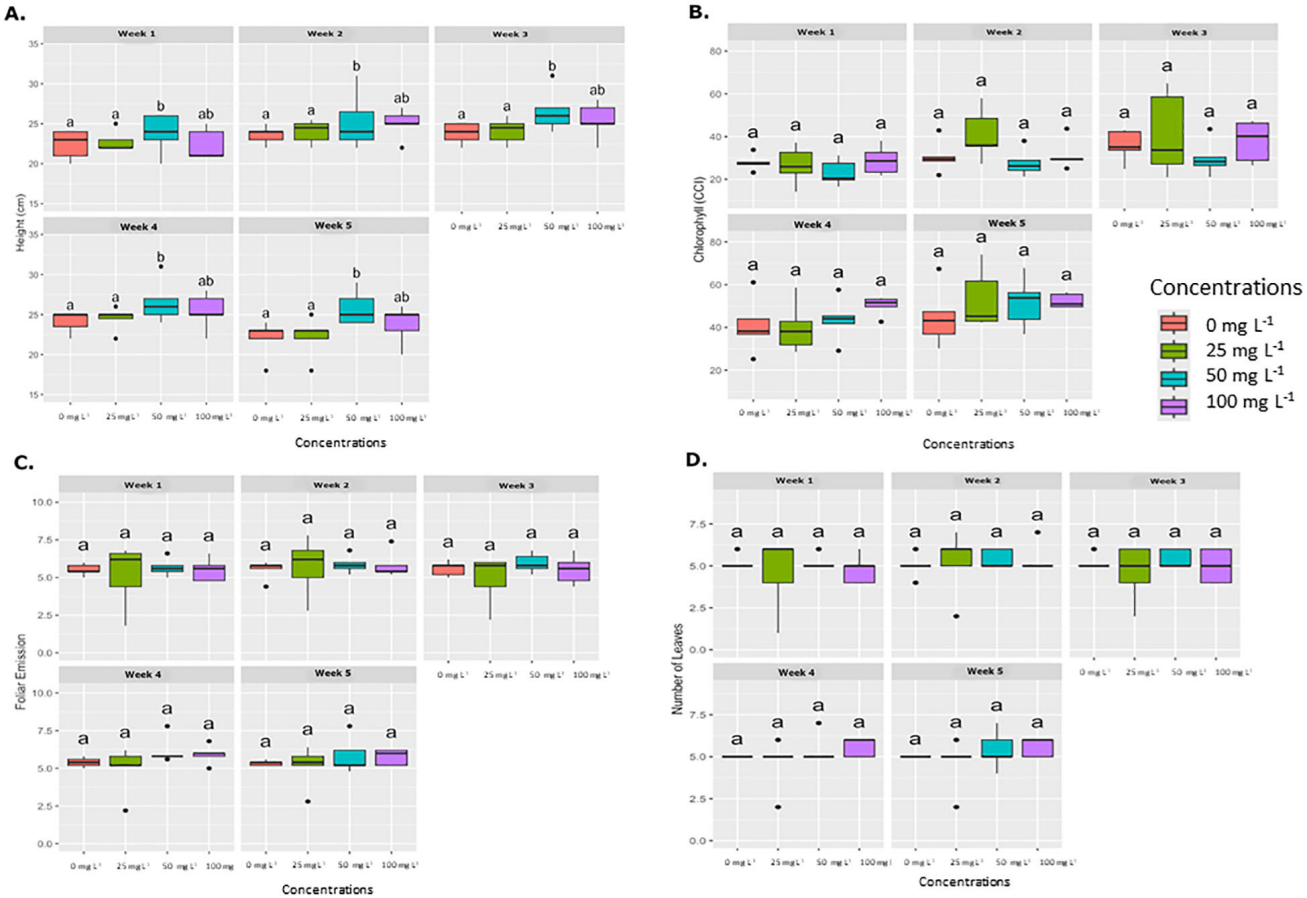
Weekly average effect of AgNPs applied by foliar on different physiological parameters. Boxplots illustrate the effects of various concentrations of AgNPs (0, 25, 50, and 100 mg L^-1^) on four physiological parameters, which are measured weekly over five weeks. **(A)** Plant height (cm), **(B)** Chlorophyll content (SPAD), **(C)** Foliar emission rate, and **(D)** Number of leaves. Significant differences between treatments within each week are denoted by different letters (a, b, ab) according to Tukey’s *post-hoc* test (p < 0.05).

**Figure 3** shows the average results for various physiological parameters measured in plants treated with the foliar application method. These parameters, including plant height (cm), foliar emission, and chlorophyll concentration (SPAD units), were evaluated weekly. No significant differences were observed in chlorophyll concentration, leaf emission, or leaf number among the different AgNP concentrations over the evaluation period. However, plant height showed statistically significant differences at AgNP concentrations of 50 and 100 mg L^−1^ compared to the control (0 mg L^−1^).

##### 3.2.1.2 Drench application

**Figure 4:**
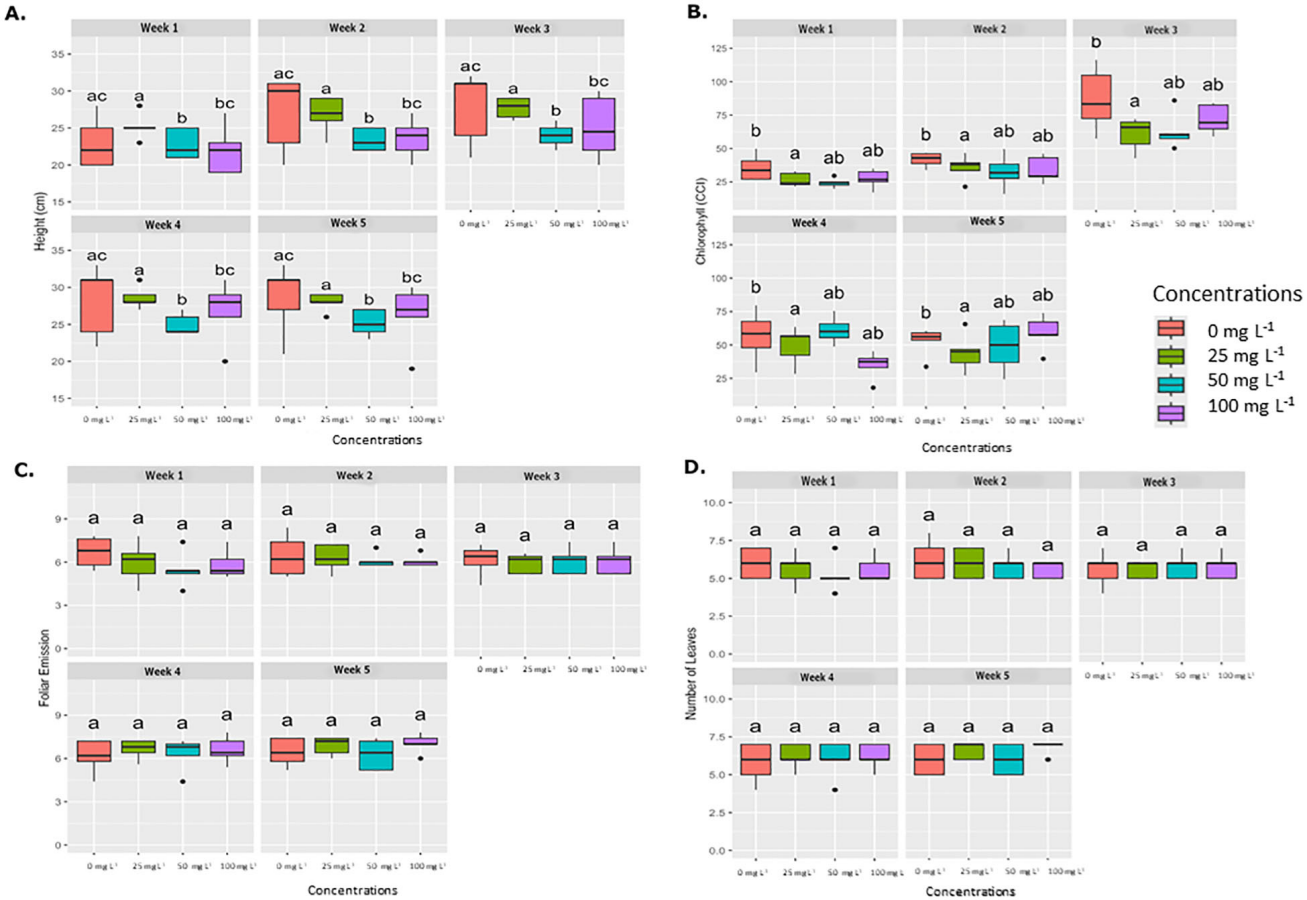
Average weekly effect of AgNPs applied by drench on different physiological parameters. Boxplots illustrate the effects of various concentrations of nanoparticles (0 mg L-1, 25 mg L-1, 50 mg L-1, and 100 mg L^-1^.) on four physiological parameters, which are measured weekly over five weeks. **(A)** Plant height (cm), **(B)** Chlorophyll content (SPAD), **(C)** Foliar emission rate, and **(D)** Number of leaves. Significant differences between treatments within each week are denoted by different letters (a, b, ab) according to Tukey’s *post-hoc* test (p < 0.05).

Similar to the foliar application results, the drench application did not present statistical significance in foliar emission or leaf number across weekly evaluation periods, as shown in **Figure 4**. However, significant differences were observed in plant height at concentrations of 50 mg L^−1^ and 100 mg L^−1^ compared to the control (0 mg L^−1^). Chlorophyll concentration (SPAD) also displayed significant differences at concentrations of 25, 50, and 100 mg L^−1^ relative to the control. Therefore, exposure to AgNPs did not have a significant impact on the development of banana plants.

Root length measurements taken at the end of the greenhouse physiological analysis showed no significant differences between AgNP-treated plants and the control group (0 mg L^−1^) for either foliar or drench application. Therefore, at three months of plant growth, no negative effect from AgNPs on root development was observed. (**Supplementary Figures S1, S2**).

#### 3.2.2 Chlorophyll a, b, and total analysis

**Figure 5:**
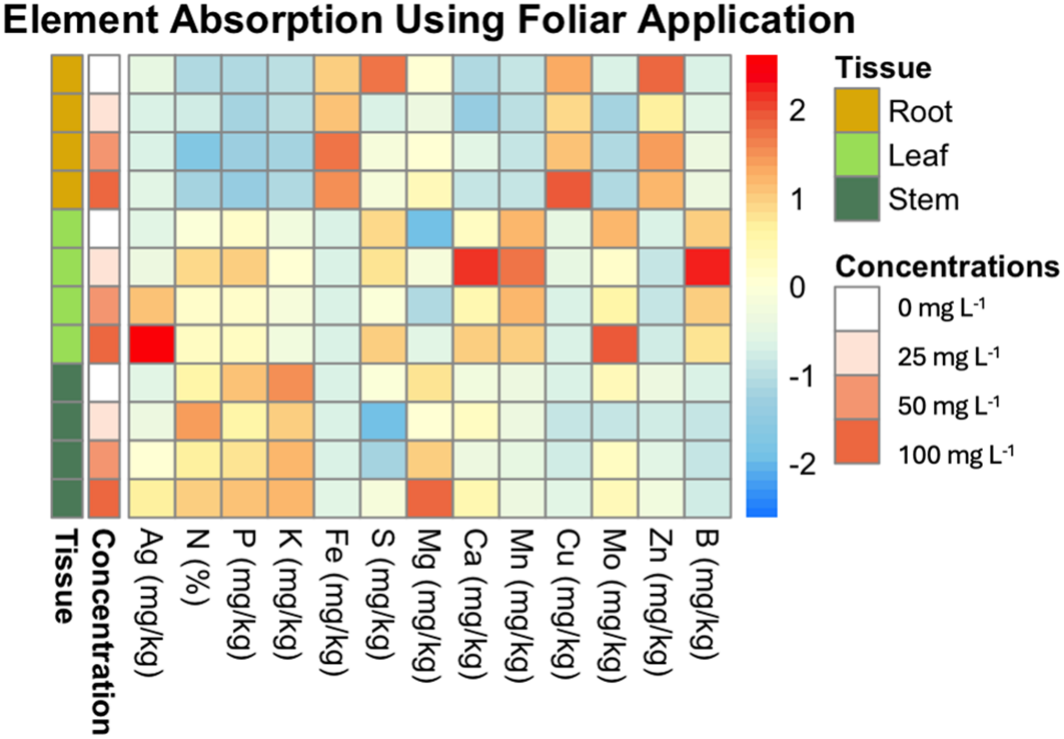
Heatmap of element absorption in plant tissues across different concentrations of AgNPs using foliar application. This heatmap visualizes the average absorption of various elements (e.g., Mg, Ag, Fe) in different plant tissues (root, leaf, stem) subjected to varying concentrations of AgNPs (0, 25, 50, and 100 mg L^-1^). The rows are ordered by concentration and tissue type. The color scale ranges from blue (low absorption) to red (high absorption), highlighting the differences in element uptake.

At the end of the experiment, samples were collected from each plant to measure chlorophyll a, chlorophyll b, and total chlorophyll under varying AgNP concentrations, applied through both drench and foliar methods. **Figure 5** presents the data distribution using a violin plot.

For chlorophyll a, the foliar application shows a more uniform distribution of values, with most data clustered around similar values, except for the control (0 mg L^−1^), which displays a distinct difference. In contrast, the drench application at 25 mg L^−1^ shows a wider range and narrower distribution, while at 50 mg L^−1^ and 100 mg L^−1^, the distribution is similar between the two concentrations (**Supplementary Figure S3**).

In contrast, for chlorophyll b, the foliar application at 100 mg L^−1^ displays a wider range of values, while the other concentrations show relatively uniform distributions. And, for total chlorophyll, the foliar application at 25, 50, and 100 mg L^−1^ presents higher values compared to the control (0 mg L^−1^), whereas the drench application shows more compact distributions across the concentrations (**Supplementary Figure S3**).

#### 3.2.3 Elemental analysis of plant tissues for Ag and mineral nutrients

##### 3.2.3.1 Foliar application

**Figure 5** illustrates the effects of AgNPs on nutrient uptake across different plant tissues, specifically roots, leaves, and stems, at AgNP concentrations of 0, 25, 50, and 100 mg L^-1^. The results reveal distinct nutrient uptake patterns across tissues and concentrations.

The data show that Zn, Cu, and Fe are most concentrated in root tissue, while Mo, Ag, Mn, B, S, and Ca are more concentrated in leaves. P, N, K, and Mg are found predominantly in stems.

For Ag, there is a clear dose-dependent increase in concentration, with significant accumulation in leaves and stems at 50 and 100 mg L^−1^, whereas roots maintained the lowest Ag levels (**Figure 5**).

For Fe and Mn, no significant differences were observed across concentrations in any tissue, indicating that AgNP exposure did not affect these elements’ uptake.

In the case of Zn, significant differences were observed in root tissue between 0 mg L^−1^ and both 25 mg L^−1^ and 50 mg L^−1^ concentrations.

Although Ca levels appeared elevated at 25 mg L^−1^ in leaves (as shown in the heatmap), no significant differences were found in any comparisons.

For Cu and Mo, there were no significant differences across concentrations in the leaf and root. However, in stems, Mo content showed a significant difference between the control and the rest of the concentrations. Cu showed significant differences between 0 mg L^−1^, and 50 mg L^−1^ in the stem.

No significant differences were found in K and Mg content in root and stem tissues across concentrations. However, in leaf tissue, K differed significantly between 0 mg L^−1^ and 25 mg L^−1^, and between 100 mg L^−1^ and 25 mg L^−1^. For Mg, significant differences were observed at 25 mg L^−1^ and 100 mg L^−1^ compared to the control (0 mg L^−1^).

For B, significant differences were observed in leaf and root tissues at certain concentrations. In leaf tissue, 25 mg L^-1^ showed the highest B content, significantly differing from the 100 mg L^-1^. In root tissue, significant differences were detected at 50 mg L^-1^ and 100 mg L^-1^ compared to the control (0 mg L^-1^).

For P, significant differences were found in leaf tissue between 0 mg L^−1^ and 25 mg L^−1^, as well as between 25 mg L^−1^ and 50 mg L^−1^. No significant differences were detected in root and stem tissues.

In terms of N content, significant differences were observed across all tissues. In leaf tissue, N was highest at 25 mg L^−1^, showing significant differences compared to 0 mg L^−1^, 50 mg L^−1^, and 100 mg L^−1^. In root tissue, a significant difference was found between 25 mg L^−1^ and 50 mg L^−1^. For stems, the N percentage at 25 mg L^−1^ was significantly different from both 0 mg L^−1^ and 50 mg L^−1^.

Lastly, S content showed significant differences in root and stem tissues, though not in leaf tissue. In stems, S content at 25 mg L^−1^ and 50 mg L^−1^ was lower than the control, while 100 mg L^−1^ showed no significant difference from the control. In roots, S content decreased significantly at 25 mg L^−1^ compared to the control.

##### 3.2.3.2 Drench application

**Figure 6:**
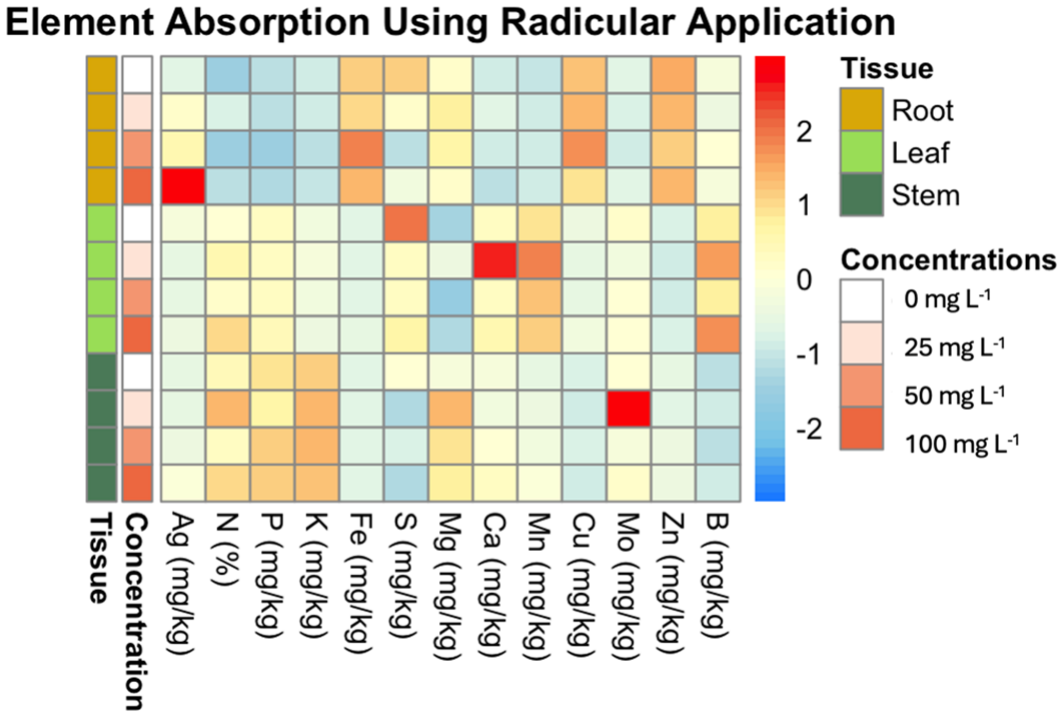
Heatmap of element absorption in plant tissues across different concentrations of AgNPs using drench application. This heatmap visualizes the average absorption of various elements (e.g., Mn, Ag, Ca) in different plant tissues (root, leaf, stem) subjected to varying concentrations of AgNPs (0 mg L^-1^, 25 mg L^-1^, 50 mg L^-1^, 100 mg L^-1^). The rows are ordered by concentration and tissue type. The color scale ranges from blue (low absorption) to red (high absorption), highlighting the differences in element uptake.

**Figure 6** illustrates the uptake ratio of each element in the plant tissues (leaf, stem, and root) at the different AgNP concentrations (0 mg L^−1^, 25 mg L^−1^, 50 mg L^−1^, 100 mg L^−1^). The heatmap reveals that Ag is most concentrated in root tissue, with Ag content increasing proportionally to nanoparticle concentration. Significant differences in Ag content were observed in root tissue between 0 mg L^−1^ and 100 mg L^−1^, as well as between 25 mg L^−1^, 50 mg L^−1^, and 100 mg L^−1^, whereas 25 mg L^−1^ and 50 mg L^−1^ did not differ significantly from the control (0 mg L^−1^). In stem tissue, only the 0 mg L^−1^ and 100 mg L^−1^ concentrations showed a statistically significant difference compared to the control.

Ca was found to be more concentrated in leaf tissue, with the highest content at 25 mg L^−1^, which significantly differed from other concentrations (0 mg L^−1^, 50 mg L^−1^, and 100 mg L^−1^). No significant differences in Ca levels were observed in root or stem tissues.

Mn was also more concentrated in leaves. At 25 mg L^−1^, the leaf tissue exhibited the highest Mn concentration, significantly differing from the 0 mg L^−1^ control.

Mo primarily accumulated in the stem tissue. However, in leaf tissue, Mo concentration peaked at 25 mg L^−1^, showing a significant difference from other concentrations (0 mg L^−1^, 50 mg L^−1^, and 100 mg L^−1^).

The drench application did not lead to significant differences in the concentrations of N, P, K, B, Cu, Fe, Zn, or Mg across plant tissues. These elements were unaffected by AgNP treatment. Root tissue had the highest concentrations of Zn, Fe, and Cu, while stem tissue showed elevated levels of K, N, and P. S was more concentrated in leaf tissue, with a noted decrease in S content as AgNP concentration increased.

### 3.3 qPCR analysis

**Figure 7:**
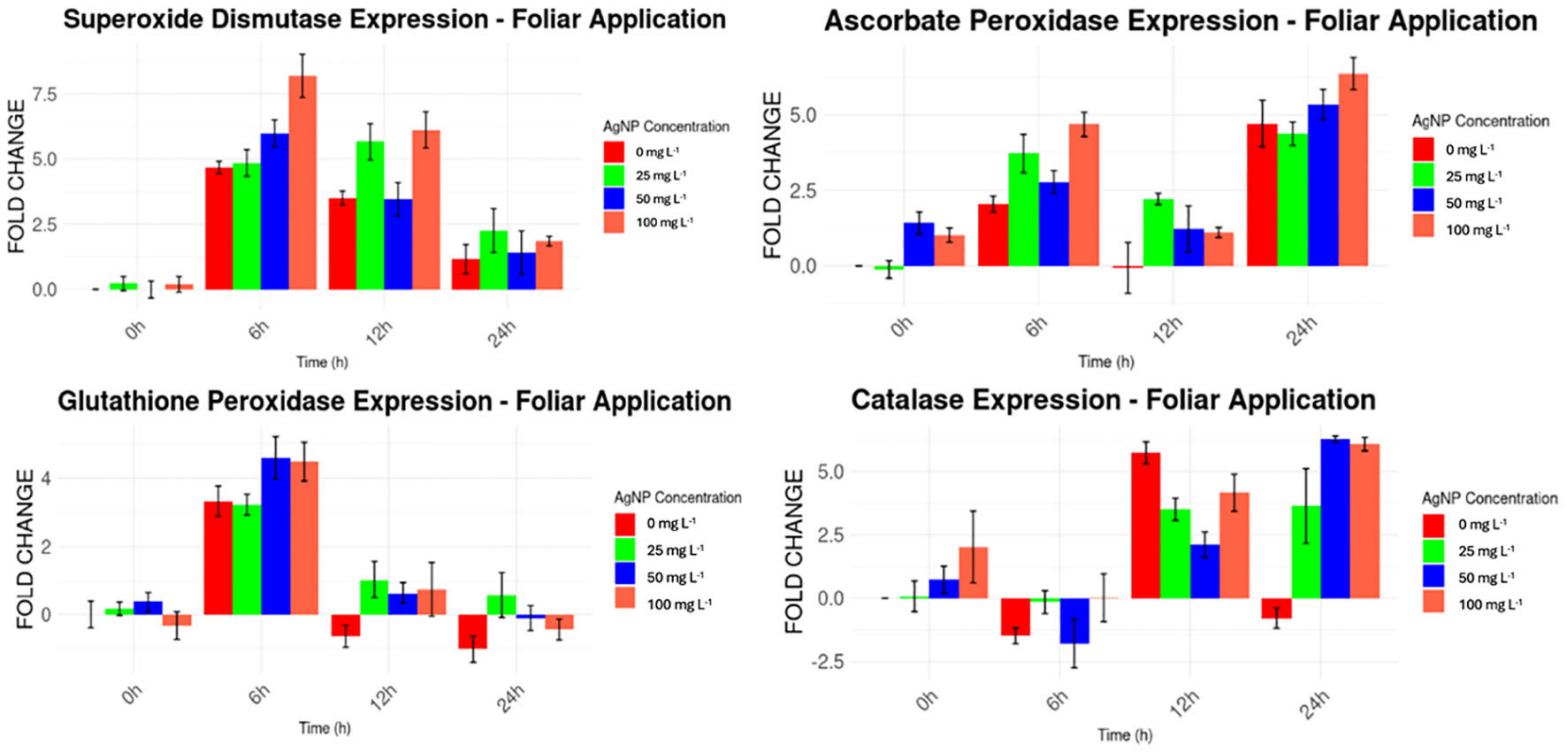
Expression levels of stress genes by foliar AgNPs application compared to the housekeeping gene.

Ct values were used to compare the expression levels of the genes of interest against the normal expression of the Actin housekeeping gene. For the foliar application of AgNPs (**Figure 7**), higher expression levels were consistently observed for *MaSOD* and *MaAPX* from the beginning of the experiment, ranging from 1 to 8 times higher than the expression of the housekeeping gene. In contrast, *MaGPX* and *MaCAT* displayed irregular expression patterns. *MaGPX* showed higher expression at 0 and 6 hours, but lower levels at 12 and 24 hours. Similarly, *MaCAT* expression was elevated at 12 and 24 hours but decreased at 6 hours, compared to the housekeeping gene.

**Figure 8:**
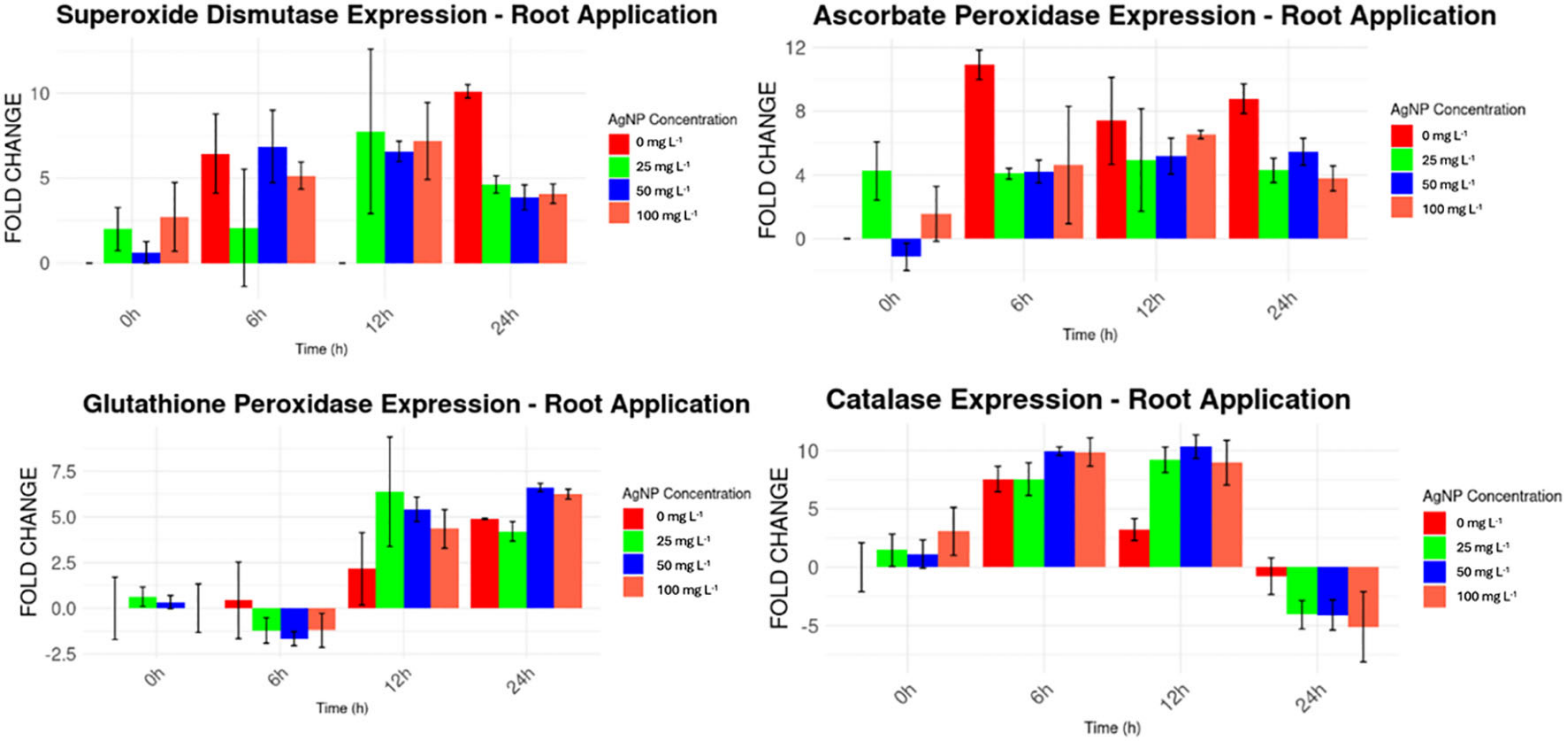
Expression levels of stress genes by drench AgNPs application compared to a housekeeping gene.

For the root application of nanoparticles (**Figure 8**), a similar pattern to the foliar application was observed. Higher expression levels were found for *MaSOD* and *MaAPX* from the beginning of the experiment, with expression levels ranging from 1 to 12 times higher than the housekeeping gene, surpassing the levels observed in the foliar application. *MaGPX* and *MaCAT*, however, showed irregular expression patterns, fluctuating from higher expression levels (e.g., *MaGPX* at 12 and 24 hours, and *MaCAT* from 0 to 12 hours) to lower levels (e.g., *MaGPX* from 0 to 6 hours, and *MaCAT* at 24 hours).

Significant differences in gene expression (p < 0.05) were observed for *MaSOD and MaAPX* at 6h and 24h, *MaGPX* at 6h, and *MaCAT*, when AgNPs were applied via foliar application (**Table 2**), compared to baseline levels at the beginning of the experiment.

**Table 2:** Levels of expression of stress genes against nanoparticles applied to leaves, analyzed versus controls taken at hour 0.

		*MaSOD*		*MaAPX*		*MaGPX*		*MaCAT*	
*Time period (hour)*	AgNP concentrations (mg L^-1^)	Fold Change	p value	Fold Change	p value	Fold Change	p value	Fold Change	p value
*0*	0	0,000		0,000		0,000		0,000	
*0*	25	-0,009		1,416		0,374		0,732	
*0*	50	0,194		1,021		-0,324		2,023	
*0*	100	0,221		-0,117		0,168		0,077	
*6*	0	4,677	0,003	2,049	0,010	3,333	0,001	-1,480	0,024
*6*	25	5,983		2,785		4,595		-1,777	
*6*	50	8,188		4,691		4,484		0,024	
*6*	100	4,846		3,724		3,231		-0,154	
*12*	0	3,508	0,003	-0,065	0,216	-0,634	0,190	5,727	0,022
*12*	25	3,457		1,229		0,644		2,106	
*12*	50	6,114		1,109		0,745		4,160	
*12*	100	5,655		2,218		1,036		3,505	
*24*	0	1,156	0,002	4,716	0,000	-1,011	0,197	-0,780	0,053
*24*	25	1,399		5,351		-0,107		6,271	
*24*	50	1,847		6,374		-0,437		6,074	
*24*	100	2,255		4,377		0,581		3,634	

For root application, significant differences were detected for *MaSOD* at 6h and 12h, *MaCAT* at 12h, *MaAPX* at 12h and 24h, and *MaGPX* at all time points (**Table 3**).

**Table 3:** Levels of expression of stress genes against nanoparticles applied to roots analyzed versus controls taken at hour 0.

		*MaSOD*		*MaAPX*		*MaGPX*		*MaCAT*	
*Time period (hour)*	AgNP concentrations (mg L^-1^)	Fold Change	p value	Fold Change	p value	Fold Change	p value	Fold Change	p value
*0*	0	0,000		0,000		0,000		0,000	
*0*	25	0,630		-1,141		0,334		1,128	
*0*	50	2,724		1,562		0,004		3,070	
*0*	100	2,002		4,251		0,635		1,451	
*6*	0	6,446	0,046	10,910	0,066	0,430	0,065	7,558	0,001
*6*	25	6,871		4,218		-1,661		9,955	
*6*	50	5,154		4,628		-1,202		9,870	
*6*	100	2,075		4,079		-1,214		7,555	
*12*	0	0,000	0,031	7,394	0,023	2,157	0,006	3,225	0,007
*12*	25	6,580		5,184		5,415		10,333	
*12*	50	7,191		6,533		4,345		8,960	
*12*	100	7,756		4,936		6,379		9,213	
*24*	0	10,118	0,057	8,787	0,057	4,897	0,002	-0,762	0,025
*24*	25	3,869		5,456		6,609		-4,094	
*24*	50	4,087		3,781		6,245		-5,104	
*24*	100	4,630		4,290		4,209		-4,070	

To determine whether gene expression changes were due to the presence of AgNPs or the application of the solvent alone, a control analysis was performed. The control groups (for both application methods, with 0 mg L^−1^ AgNPs at each sampling time) were treated only with the solvent, without AgNPs, from 6 to 24 hours.

In the root application samples (**Table 4**), only one instance of a significant difference in stress gene expression was found between samples with AgNPs and those with the solvent alone. This difference was observed for *MaAPX* at 25 mg L^−1^, indicating an effect attributable to AgNPs rather than the solvent (Triton X).

**Table 4:** Gene expression of stress-related genes under the application of nanoparticles applied to the roots within different time points.

Gene	Sample	Fold Change	Sample	Fold Change	Sample	Fold Change	Sample	Fold Change
*MaSOD*	R-0-0	0.000	R-0-25	0.630	R-0-50	2.724	R-0-100	2.002
	R-6-0	6.446	R-6-25	6.871	R-6-50	5.154	R-6-100	2.075
	R-12-0	0.000	R-12-25	6.580	R-12-50	7.191	R-12-100	7.756
	R-24-0	10.118	R-24-25	3.869	R-24-50	4.087	R-24-100	4.630
			p Value	0.471	p Value	0.433	p Value	0.480
*MaAPX*	R-0-0	0.000	R-0-25	-1.141	R-0-50	1.562	R-0-100	4.251
	R-6-0	10.910	R-6-25	4.218	R-6-50	4.628	R-6-100	4.079
	R-12-0	7.394	R-12-25	5.184	R-12-50	6.533	R-12-100	4.936
	R-24-0	8.787	R-24-25	5.456	R-24-50	3.781	R-24-100	4.290
			p Value	0.034	p Value	0.120	p Value	0.195
*MaGPX*	R-0-0	0.000	R-0-25	0.334	R-0-50	0.004	R-0-100	0.635
	R-6-0	0.430	R-6-25	-1.661	R-6-50	-1.202	R-6-100	-1.214
	R-12-0	2.157	R-12-25	5.415	R-12-50	4.345	R-12-100	6.379
	R-24-0	4.897	R-24-25	6.609	R-24-50	6.245	R-24-100	4.209
			p Value	0.265	p Value	0.304	p Value	0.328
*MaCAT*	R-0-0	0.000	R-0-25	1.128	R-0-50	3.070	R-0-100	1.451
	R-6-0	7.558	R-6-25	9.955	R-6-50	9.870	R-6-100	7.555
	R-12-0	3.225	R-12-25	10.333	R-12-50	8.960	R-12-100	9.213
	R-24-0	-0.762	R-24-25	-4.094	R-24-50	-5.104	R-24-100	-4.070
			p Value	0.229	p Value	0.243	p Value	0.315

For foliar application (**Table 5**), three instances of significant differences were noted between samples with AgNPs and those with the stabilizer alone (PVP). These differences were observed in *MaAPX* at 25 mg L^−1^ and 50 mg L^−1^, and in *MaGPX* at 25 mg L^−1^, indicating that the AgNPs had a distinct effect on stress gene expression compared to the stabilizer.

**Table 5:** Gene expression of stress-related genes under the application of nanoparticles applied to leaves within different time points.

Gene	Sample	Fold Change	Sample	Fold Change	Sample	Fold Change	Sample	Fold Change
*MaSOD*	H-0-0	0.000	H-0-25	-0.009	H-0-50	0.194	H-0-100	0.221
	H-6-0	4.677	H-6-25	5.983	H-6-50	8.188	H-6-100	4.846
	H-12-0	3.508	H-12-25	3.457	H-12-50	6.114	H-12-100	5.655
	H-24-0	1.156	H-24-25	1.399	H-24-50	1.847	H-24-100	2.255
			p value	0.163	p value	0.056	p value	0.073
*MaAPX*	H-0-0	0.000	H-0-25	1.416	H-0-50	1.021	H-0-100	-0.117
	H-6-0	2.049	H-6-25	2.785	H-6-50	4.691	H-6-100	3.724
	H-12-0	-0.065	H-12-25	1.229	H-12-50	1.109	H-12-100	2.218
	H-24-0	4.716	H-24-25	5.351	H-24-50	6.374	H-24-100	4.377
			p value	0.007	p value	0.011	p value	0.136
*MaGPX*	H-0-0	0.000	H-0-25	0.374	H-0-50	-0.324	H-0-100	0.168
	H-6-0	3.333	H-6-25	4.595	H-6-50	4.484	H-6-100	3.231
	H-12-0	-0.634	H-12-25	0.644	H-12-50	0.745	H-12-100	1.036
	H-24-0	-1.011	H-24-25	-0.107	H-24-50	-0.437	H-24-100	0.581
			p value	0.010	p value	0.082	p value	0.086
*MaCAT*	H-0-0	-0.545	H-0-25	0.732	H-0-50	2.023	H-0-100	0.077
	H-6-0	-1.480	H-6-25	-1.777	H-6-50	0.024	H-6-100	-0.154
	H-12-0	5.727	H-12-25	2.106	H-12-50	4.160	H-12-100	3.505
	H-24-0	-0.780	H-24-25	6.271	H-24-50	6.074	H-24-100	3.634
			p value	0.327	p value	0.136	p value	0.475

## 4 Discussion

### 4.1 Effect of AgNPs concentrations on *in vitro* assays in Cavendish banana

The study assessed the effects of AgNPs on banana cultivars *in vitro*, using doses similar to those employed in our previous research on antifungal activity against *Fusarium oxysporum* f.sp. *cubense* (Mendoza et al., 2025). The selected doses, effective in pathogen control, were tested here to evaluate their impact on plant physiology before scaling to greenhouse conditions.

*In vitro* evaluation was essential to detect early phytotoxicity of AgNPs under controlled conditions, minimizing external variables before exposing plants to more complex conditions. Our findings showed that AgNPs negatively impact various physiological parameters in the examined plants, including leaf count, shoot length, chlorophyll content index (CCI), root count, and root length.

Statistically significant differences were observed between AgNP-treated samples and the control group. Notably, chlorophyll content in AgNP-supplemented plants was reduced by over 50% in all the concentrations, which is particularly significant as high chlorophyll levels are essential for maximizing photosynthetic efficiency, promoting robust growth and development, enhancing stress resistance, and ultimately boosting crop yields (Slattery et al., 2017). This reduction thus serves as a crucial indicator of compromised plant health (Slattery et al., 2017).

Furthermore, a concentration of 1000 mg L^−1^ proved lethal to the banana explants within two weeks, completely inhibiting root formation. While AgNPs have been reported to be effective at much lower concentrations (Do et al., 2018), such high concentrations appear detrimental to plant viability. Additionally, AgNPs have been explored as disinfectants for vegetative explants, demonstrating antimicrobial efficacy comparable to traditional sterilizing methods such as NaClO, H_2_O_2_, HgCl_2_ (Mo et al., 2020; Tung et al., 2021; Urnukhsaikhan et al., 2021).

AgNPs-treated samples demonstrated that concentrations of 25, 50, and 100 mg L^-1^ did not enhance root formation or development compared to the control (0 mg L^-1^). A study on the impact of AgNPs on root development in *Allium cepa* reported a reduction in mitochondrial activity in the root tips exposed to AgNPs (≤100 nm in size) at concentrations of 25, 50, 75 and 100 mg L^-1^ for 4h (Noori et al., 2024). In contrast, similar studies have shown that *in vitro* culture media with 3 mg L^-1^ of AgNPs resulted in improved root number and length, whereas increasing he concentration to 7 mg L^−1^ limited explant (Do et al., 2018). These findings align with the effects observed in this study at higher concentrations.

### 4.2 Greenhouse assays in Gros Michel banana

#### 4.2.1 Physiological analysis

Physiological analysis revealed that AgNPs applied by drench or foliar application did not negatively impact chlorophyll content, leaf emergence, or leaf number in banana plants at any of the tested concentrations. However, plant height was the only parameter that showed a statistically significant difference at concentrations of 50 mg L^-1^ and 100 mg L^-1^.

Currently, no studies have examined the interaction of AgNPs with banana plants under greenhouse conditions. However, a study conducted in Egypt reported that higher concentrations (e.g., 200 mg L^-1^) inhibited plant growth, highlighting the importance of carefully managing AgNP concentrations when applying them to crops (El-Mahdy et al., 2019).

Interestingly, AgNPs did not reduce chlorophyll content in greenhouse plants, despite the significant reduction observed in *in vitro* assays. This difference may indicate that soil interactions with AgNPs may influence plant uptake, reducing AgNP bioavailability or enabling plants to activate recovery mechanisms over a longer growth period (Cycoń et al., 2019).

Given that 200 mg L^-1^ was previously shown to be harmful, while 50–100 mg L^-1^ in this study only affected plant height. These findings indicate that AgNPs at these concentrations do not severely impair plant development. Notably, Argovit AgNPs have been tested in field conditions for their effectiveness against *Huanglongbing* disease in Mexican lime (*Citrus aurantifolia* Swingle) (Stephano-Hornedo et al., 2020). In that study, AgNPs were not affected by light exposure, which enables their use under light-exposed conditions. Based on this, we assume that once AgNPs enter plant tissues, they remain stable and are not significantly influenced by light.

However, further studies are needed to evaluate the long-term effects of AgNPs exposure, including potential interactions with environmental factors such as soil microbiota, temperature, humidity, soil pH, and water quality, to ensure their safe and sustainable application in banana cultivation. Additionally, a comparison of this AgNP formulation with other types of nanoparticles and conventional antifungal agents is planned for future studies to better contextualize its efficacy and safety in agricultural systems.

#### 4.2.2 Effect of AgNPs on the uptaking of elements and tissue distribution in the plants

**Figure 5** presents key insights into the effects of AgNP foliar application on nutrient uptake in banana plants. The data indicate that AgNPs influence the nutrient distribution across plant tissues. Notably, elements such as Zn, Cu, and Fe are more concentrated in the roots, while Mo, Ag, Mn, B, and Ca are more concentrated in the leaves. Increasing AgNP concentrations led to a progressive rise in Ag accumulation, with the highest levels observed in leaves, followed by stems and roots.

Foliar AgNP application also increased Mg concentration, particularly in the leaves, as well as N (%) in all three tissues, with the most pronounced rise in N observed at a concentration of 25 mg L^-1^. In contrast, AgNP application reduced the levels of elements such as Mo and Zn, particularly at.25 mg L^-1^ and 50 mg L^-1^.

Drench application of AgNPs had a more localized effect, primarily influencing fewer nutrients. S concentration decreased across all tissues, whereas Ag accumulation increased significantly, particularly in the roots, as shown in **Figure 6**. Additionally, Ca and Mn concentrations were higher in leaves at 25 mg L^-1^, while Mo showed accumulation in stems and leaves at this concentration. Other elements, such as N, P, K, B, Cu, Fe, Zn, and Mg, remained largely unaffected.

Interestingly, leaves and stems exhibited the highest element uptake under foliar application, whereas the roots showed greater element accumulation under drench application. This suggests that the method of AgNP application directly influences nutrient distribution across plant tissues. The relation between AgNP concentration and Ag content across tissues underscores the necessity of understanding the mechanistic pathways by which AgNPs interact with plant physiology

A study on the physiological responses of plants treated with AgNPs suggests that AgNPs can be transported into plant tissues due to their nanoscale size, typically between 1 and 100 nm (Anjum et al., 2013). By comparison, the dimensions of plasmodesmata and the pores within plant cell walls generally range from 5 to 20 nm, potentially allowing for nanoparticle passage (Carlson et al., 2008). Although the precise mechanisms of AgNP transport within plant cells are still under investigation, one proposed pathway involves the use of potassium (K^+^) channels and copper (Cu^2+^) transporters for AgNP delivery (Mueller and Nowack, 2008; Navarro et al., 2008; Leonardo et al., 2016). Notably, the application of AgNPs does not appear to disrupt K and Cu levels, suggesting that AgNPs may utilize these transporters without interfering with the physiological balance of these essential nutrients.

Another study conducted in Brazil emphasizes the high nutrient demands of banana plants, especially for N, P, K, Ca, Mg, and S, to maintain healthy growth and productivity throughout the cultivation cycle (de Deus et al., 2020). The nutritional analysis results indicated that AgNPs did not significantly affect the concentrations of K, Ca, and Mg when applied via foliar or drench, suggesting that AgNPs have low toxicity to the plant. These nutritional requirements, particularly for N and P, are critical for supporting the plant’s metabolic functions and yield potential.

The nutritional analysis in this study indicated that AgNPs, whether applied via foliar spray or drench, did not significantly affect the concentrations of K, Ca, and Mg, suggesting that AgNPs have a low toxicity profile for these essential elements. However, a notable decrease in sulfur content was observed with increasing AgNP concentrations when applied through drenching. Sulfur is essential for plant metabolism, as it is a component of amino acids and plays a critical role in protein synthesis, influencing photosynthesis, respiration, and nitrogen fixation (de Deus et al., 2020). The effects on P and N content, however, were not substantial, indicating a selective impact of AgNPs on nutrient uptake.

Overall, AgNPs at concentrations of 25 mg L^-1^ and 50 mg L^-1^ influenced nutrient absorption, with the most variability observed in the leaves and stems under foliar application and in the roots under drench application. While some elements exhibited significant uptake changes, others remained unaffected, indicating a complex interaction between AgNP concentration and nutrient dynamics. Additional studies are necessary to fully understand the long-term implications of AgNP exposure on nutrient uptake, metabolic functions.

Nanoparticle size is a critical factor to consider when evaluating AgNP–plant interactions, as it directly influences uptake, translocation, and potential toxicity (Cho et al., 2018; Perde-Schrepler et al., 2019). In this study, the AgNPs applied were smaller than 50 nm, consistent with those used in our previous research, where they demonstrated effective antifungal activity. Smaller particles exhibit higher surface-area-to-volume ratios and greater reactivity, which have been linked to increased oxidative stress and cellular damage in plant and animal systems.

For instance, Cho et al. (2018) observed that 10 nm AgNPs caused significantly more acute toxicity in mice compared to 60 and 100 nm particles, highlighting the importance of particle size in nanotoxicological responses. Although the AgNPs used here did not disrupt key macronutrient levels such as K, Ca, or Mg, the observed changes in micronutrient uptake suggest the need for further optimization. Future studies should be done to optimize AgNP formulations by considering factors such as stabilizer concentration, nanoparticle size, and other physicochemical properties.

### 4.3 qPCR analysis

The toxicity of AgNPs is closely linked to oxidative stress, stemming from the generation of excessive reactive oxygen species (ROS) following exposure to AgNPs (Yan and Chen, 2019). Plant cells engage a series of antioxidant defense mechanisms to counteract the harmful impact of ROS. These mechanisms rely on the induction and actions of various genes, including *MaSOD*, *MaCAT*, and *MaAPX* (Tripathi et al., 2017). At the molecular level, the expression changes of genes that are associated with the response to AgNPs may underlie the antioxidant defense mechanisms of plants in response to AgNPs (El-Mahdy et al., 2019). In this study the exposure to AgNPs has been shown to alter the expression levels of genes associated with the stress response, suggesting a connection between exposure to AgNPs and changes in gene expression related to antioxidant defense mechanisms in plants, although other external factors cannot be ruled out, such as the diluent used, which by itself also increased the expression levels of stress genes compared to control levels.

In samples taken from plants where AgNPs were applied to the roots, a significant difference in the expression level of the stress gene *MaAPX* was observed at an AgNP concentration of 25 mg L^-1^ compared to levels in plants treated only with the solvent (Triton X). In contrast, when AgNPs were applied to the leaves, significant differences in stress gene expression were observed in three cases: *MaAPX* at AgNP concentrations of 25 and 50 mg L^-1^, and *MaGPX* at 25 mg L^-1^, relative to stress levels induced by the stabilizer (polyvinylpyrrolidone, PVP). These findings highlight the effects of AgNP exposure on gene expression and underscore the importance of considering concentration, application site, and diluent used in evaluating the impact of AgNPs on plant stress responses.

Environmental stress factors such as drought, high temperatures, salinity, and increased CO_2_ levels impact plant growth. In response, plants have developed adaptive strategies to cope with these stresses by modulating cellular and molecular activities (Ahuja et al., 2010). Numerous genes, proteins, and enzymes play roles in imparting resistance or are regulated in response to environmental stressors, including those examined in this study (Chi et al., 2019). However, some variability in stress gene expression was observed in this trial. Despite being conducted in a greenhouse under controlled conditions, extreme regional temperatures and unusually high UV radiation levels during the trial may have influenced plant stress responses. These climatic factors likely contributed to stress peaks, especially during midday, as observed in samples taken six hours after nanoparticle application.

Despite the lack of significant differences in plant growth under greenhouse conditions, the observed Ag accumulation in leaf tissues and the altered expression of antioxidant genes suggest that plants were experiencing and managing oxidative stress at the molecular level. This supports the idea that AgNPs can activate internal defense mechanisms, particularly enzymatic antioxidants such as SOD, CAT, APX, and GPX, without causing immediate morphological effects (Rao et al., 2025). AgNPs at low to moderate concentrations have been reported to function as stress-priming agents, inducing controlled ROS production and activating tolerance pathways (Fallah et al., 2024).

Additionally, the soil environment and microbial communities present in greenhouse conditions may buffer potential toxicity, as microbial interactions are known to influence the behavior and bioavailability of AgNPs in the rhizosphere, potentially reducing their phytotoxic effects (Zhai et al., 2016). This combination of molecular defense and environmental buffering may explain how banana plants in our study tolerated Ag accumulation without measurable growth inhibition, highlighting the importance of gene expression as an early indicator of plant stress.

Furthermore, the profile of Argovit™ AgNP formulations has previously been compared to silver nitrate (AgNO_3_) using the *Allium cepa* model (Casillas-figueroa et al., 2020), showing significantly lower cyto- and genotoxicity, suggesting that the effects observed in this study are likely due to nanoparticle-specific mechanisms rather than silver ions.

## 5 Conclusion

This study demonstrates that the doses of AgNPs tested, which were previously shown to be effective as an antifungal against *Fusarium oxysporum* (accepted for publication in PLOS ONE), have varying impacts on banana plants depending on their developmental stage. Smaller plants showed higher toxicity compared to larger plants. Notably, in 3-month-old plants, no negative effects on physiological parameters were detected, suggesting that the concentrations used are safe for this growth stage. The results confirm that these concentrations could serve as an effective antifungal protection dose, making them suitable for field applications.

It was also noteworthy that the plants were able to translocate the silver nanoparticles to different organs, suggesting that AgNPs could be used not only for controlling soil pathogens but also for foliar diseases. This opens up new possibilities for investigating and managing a broader range of plant diseases.

Additionally, this study sheds light on the interaction of AgNPs with essential nutrients. While AgNPs at concentrations of 25 and 50 mg L^-1^ influenced the absorption of certain elements like Mg and S, other nutrients, such as K, Ca, and N, remained unaffected. This selective impact on nutrient uptake reveals a nuanced interaction that requires careful management of AgNP concentrations to avoid adverse effects on plant health.

The molecular analysis revealed that AgNP exposure modulates stress-related gene expression, with distinct changes depending on the application site and concentration. Notably, significant upregulation of antioxidant genes like *MaAPX* and *MaGPX* suggests that AgNPs trigger stress responses, which may contribute to the observed physiological changes. This finding highlights the importance of considering both nanoparticle concentration and delivery method in the application of nanomaterials to crops, particularly under variable environmental conditions.

However, for successful field use, further research is needed to refine the synthesis of AgNPs, ensuring more stable interactions of the silver component and reducing photosensitivity. This will enhance their effectiveness in disease control across diverse environmental conditions. Moreover, future research should assess the long-term effects of AgNPs on plant health, productivity, and their environmental impact to ensure sustainable agricultural use.

## Data Availability

The original contributions present in the study are included in the article/**Supplementary Material**. Futher inquires can be directed to the corresponding author.
