# Supplementary figures and images for: Effects of silver nanoparticles on the physiology, stress, and mineral uptake of banana cultivars in vitro and greenhouse
Source: Front Plant Sci. 2025 Aug 12;16:1527137. doi: 10.3389/fpls.2025.1527137 (PMC12378748; doi:10.3389/fpls.2025.1527137)

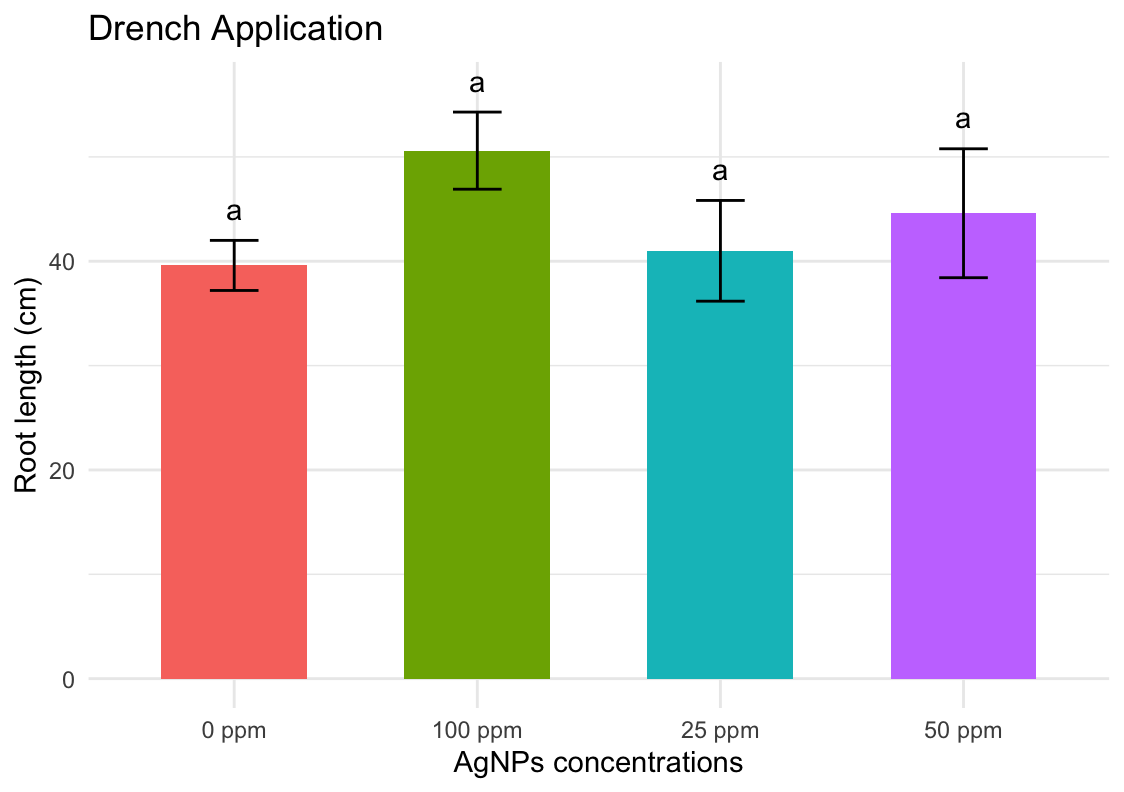

Supplement: Supplementary file 1 [file Image1.tiff]

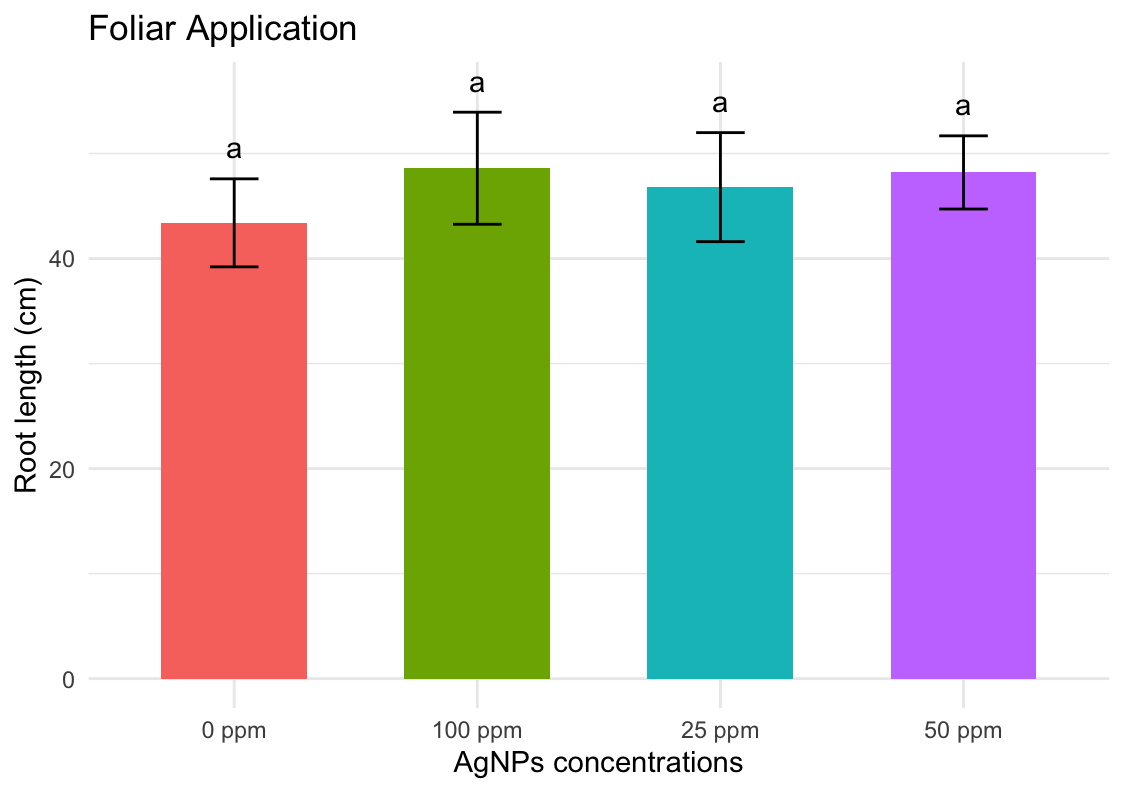

Supplement: Supplementary file 2 [file Image2.tiff]

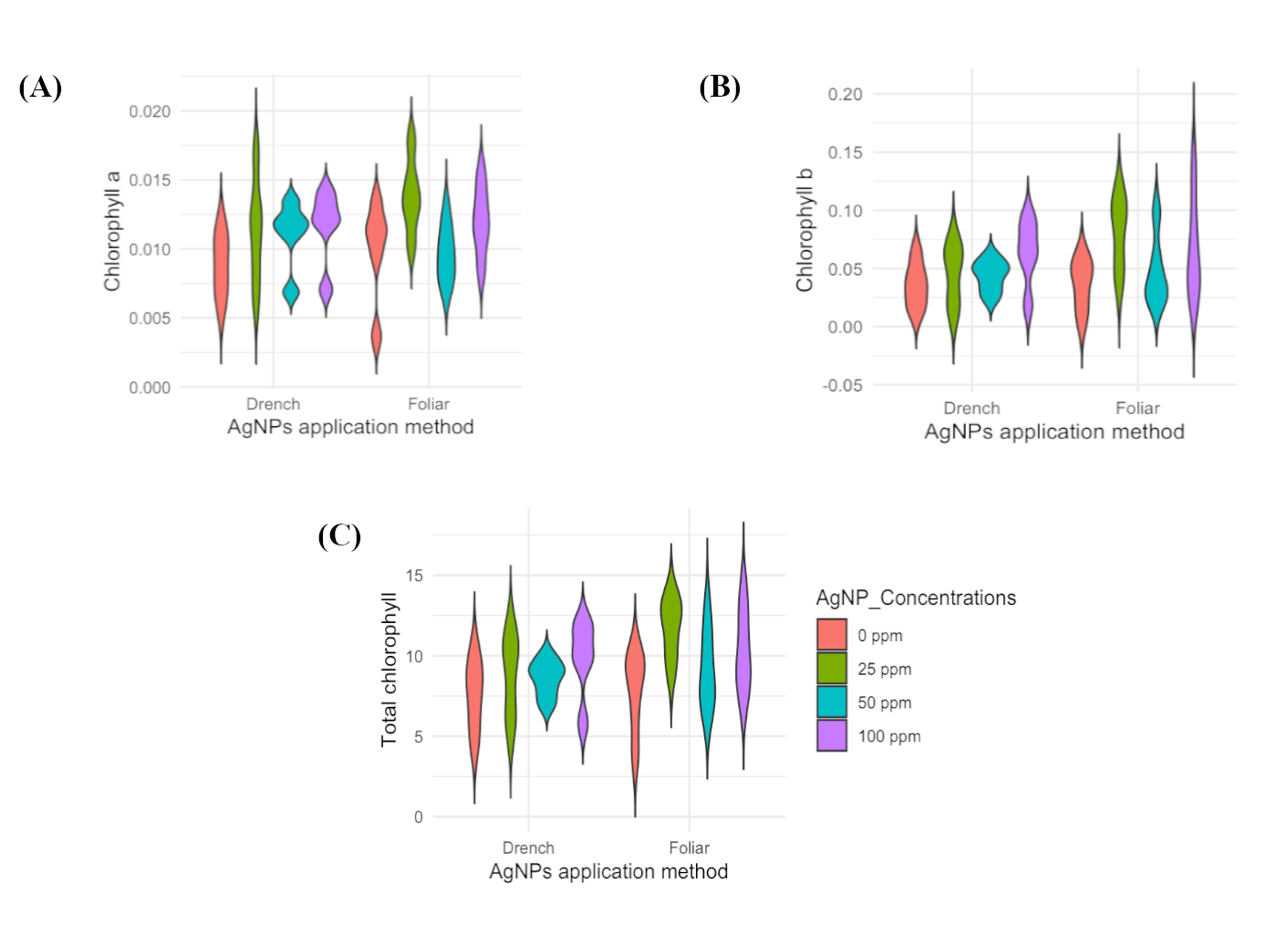

Supplement: Supplementary file 3 [file Image3.tif]

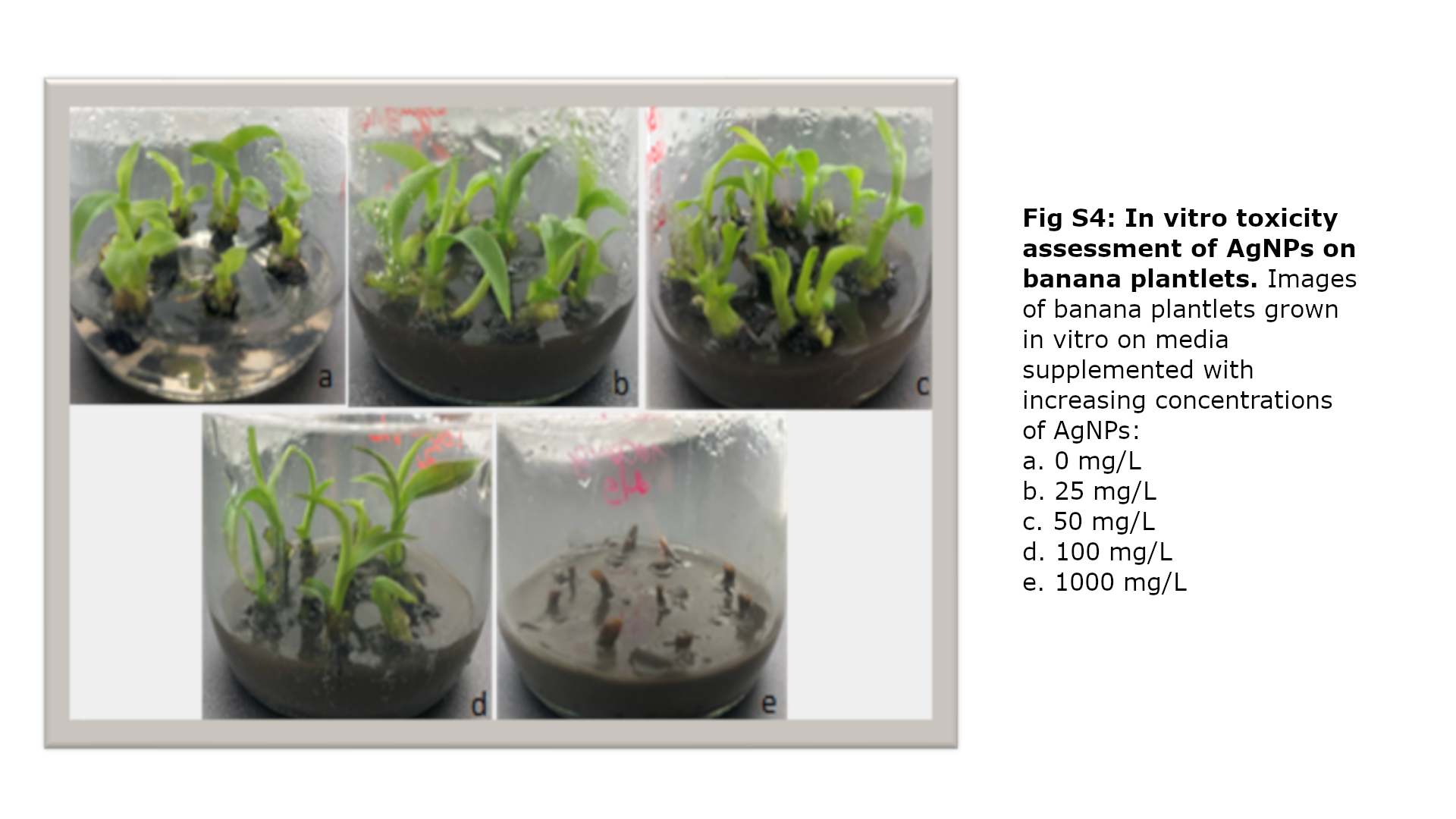

Supplement: Supplementary file 4 [file Image4.tif]

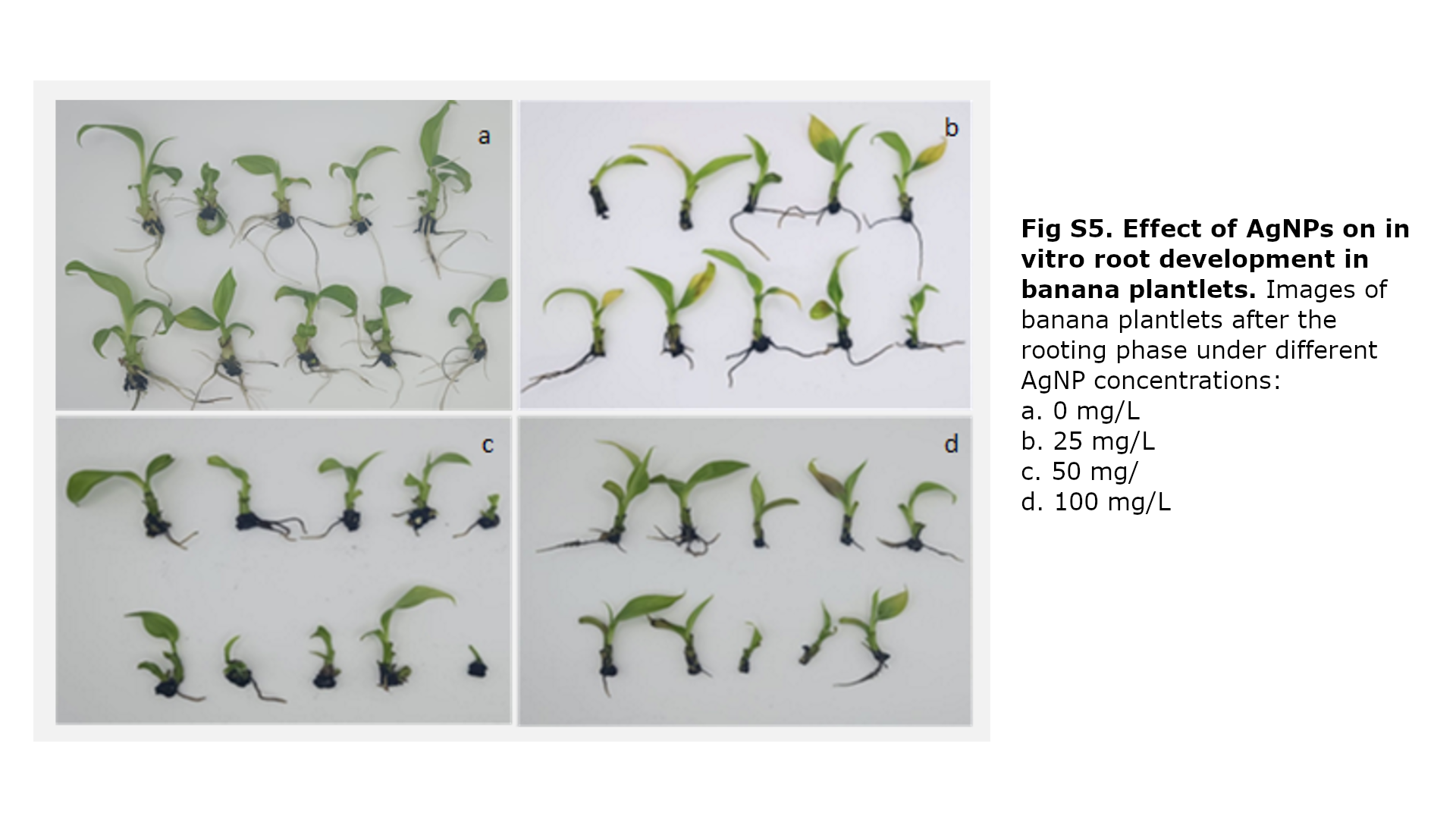

Supplement: Supplementary file 5 [file Image5.tif]
