# Supplementary material for: Effects of silver nanoparticles on the physiology, stress, and mineral uptake of banana cultivars in vitro and greenhouse
Source: Front Plant Sci. 2025 Aug 12;16:1527137. doi: 10.3389/fpls.2025.1527137 (PMC12378748; doi:10.3389/fpls.2025.1527137)
Supplement: Supplementary file 13 [file DataSheet1.pdf]

Table S1. The origin of the stabilizers and physicochemical properties of AgNP samples studied in the present work

Sample	AgNP formulation	Stabilizer		Content, wt. %		Hydrodynamic diameter, nm	Z potential, mV	AgNP characteristics according to HRTEM data**		Peak positions in UV-Vis nm
		Manufacturer	Type	Ag	Stabilizer			The average diameter, nm	Morphology	
Argovit 1220	Argovit™	Boai NKY Pharmaceuticals Ltd., (Jiaozuo, China)	PVP* with glucose additive	1.2	18.8	141.0	+12.4	Bimodal distribution with maxima at 8 nm and 80 nm	8 nm spheroidal, 80 nm pyramidal	412 and 437

* PVP is polyvinylpyrrolidone

**HRTEM data was taken from the previous publication of our group (Cruz-Ramírez et al., 2021)

Reference

Cruz-Ramírez, O. U., Valenzuela-Salas, L. M., Blanco-Salazar, A., Rodríguez-Arenas, J. A., Mier-Maldonado, P. A., García-Ramos, J. C., et al. (2021). Antitumor activity against human colorectal adenocarcinoma of silver nanoparticles: Influence of [ag]/[pvp] ratio. *Pharmaceutics* 13, 8–10. doi: 10.3390/pharmaceutics13071000
